# On Congestion of the Pulp

**Published:** 1877-05

**Authors:** George T. Barker


### ARTICLE VI.

### *On Congestion of the Pulp.*

BY PROFESSOR GEORGE T. BARKER, D. D. S.

Probably one of the most frequent pathological conditions which presents in dental practice, is that to which I have given the above title, and its very frequency, as a sequence of dental operations, makes it imperative that we should have a clear idea of the etiology, pathology, and therapeutical treatment indicated.

*Causes.*—These are both exciting and predisposing. Thus, mechanical or chemical abrasion may induce this condition, by exposure of a surface of dentine to the action of the fluids or solids taken into the mouth, or formed therein, or generated as the result of chemical solution, which, producing irritation of nerve fibrilla develops the disease. Probably the most exciting cause of congestion of the pulp is the exposure of portions of dentine to the action of substances fluid or solid, after the removal of decay, or the introduction of a metallic filling, or the action of dental caries, and it may also be induced by mechanical violence, but, when thus occasioned, it soon terminates in inflammation, peridontitis, periostitis of some writers. Another may be adverted to, viz., oxychloride of zinc fillings, which by chemical union, for some days after introduction, with particularly one of the elements of tooth structure, water, induces the morbid condition.

Imperfect occlusion of a tooth may develop congestion of the pulp, particularly if there is any existing predisposition arising from modification of the blood stream from syphilis, rheumatism, anæmia, hysteria, gout, scrofula or general debility. Of the last-named condition, I would say, that almost the first symptom of mal-nutrition is congestion of not one, but several pulps, and the patient speaks of what he calls neuralgic pains in face, in teeth, in eyes and head, flitting from one part to another, without regularity, and of great intensity. Thermal changes, by induction of elevated or depressed temperature to the pulp, through the medium of exposed tooth surface, or a good conductor, as a metallic filling, is doubtless the most fruitful cause of the disorder under consideration.

Clasps on dental substitutes, fitting closely around teeth, may, by interference with independent motion of such teeth in their sockets, establish congestion of pulp, but here, as from mechanical violence, it soon terminates in either structural degeneration, death of pulp, periostitis, alveolar abscess, or more rarely, exostosis of cementum. When the last-named condition results, the congestion of pulp may be indefinitely prolonged.

The morbid condition may also result from application of agents to obtund sensitive dentine, or long-continued use of certain remedies, as the mineral acids, the alkalies, mercury, iodine, and many other potent remedies, or from exposure to damp climate, or to very cold winds blowing upon the face.

*Symptoms.*—It may be truly said, that in the earlier stages there is but one diagnostic sign, viz., *pain*, and this pain it is extremely difficult for the patient to locate. This is not universally the case, but commonly, when several teeth have been filled, the patient states that while eating, or shortly afterwards, severe pain occurs upon one side, extending in different parts, and lasting fifteen or twenty minutes. In some there is no particular sensitiveness of any teeth to hot or cold fluids, but in others either one or the other will induce the paroxysm of pain. In the early stages there is no elongation or protusion of tooth from the socket, as that result only occurs when the congestion has passed into that of active inflammation, peridontitis therefore, while mal-occlusion, or gentle tapping or pushing of a tooth in its socket, will aid in the diagnosis, if peridontitis has been established, it will not aid in developing an irritated or congested pulp.

Occasionally the patient will indicate a particular tooth that feels full, and which he is certain is the one diseased—generally an incisor lower or perhaps a sound tooth ; therefore these statements should never influence the diagnosis, as the pain in the part is simply *reflex*, and depends for its expression on a distant irritant. In other cases the patient will indicate the tooth affected, and will describe the full feeling, and will add, "the pain usually commences and ends in that tooth." If the tooth thus pointed out has been subjected to any of the influences referred to in this paper, it is well to give the statement appropriate weight. If throbbing pain continues in any tooth for ten or fifteen minutes after application of either hot or cold fluids, it may be safely assumed that congestion of the pulp is present, and that, unless relieved, disorganization of pulp may occur, with its usual sequence before referred to. The gum structures usually show no signs of inflammation, and therefore do not aid in forming diagnosis.

*Pathology.*—Hyperæmia, diminished flow of blood in capillary vessels, which vessels are overcrowded with blood corpuscles, which pressing upon nerve fibrilla, interfere with their functions, hence the intermittent pain. There is also a tendency to effusion, and the plasma elaborated may, with the leucocytes and active cell elements of the pulp, be converted into one or more "pulp nodules," or may constitute the condition which we recognize as recession and calcification of the pulp. In all such pathological conditions, congestion of the pulp must occur, and the sequence is frequently seen in the abraded teeth of old persons, where a large portion of the pulp cavity is obliterated. In all cases of active congestion of the pulp, it must be remarked that there is a strong tendency to *stasis* of the blood from constriction of the engorged blood vessels at the appicial foramen, followed by disorganization of pulp tissue; or the congestion may, by contiguity of structure, involve the peridontium, and pass into acute inflammation of that membrane.

*Treatment.*—Immediate removal of primary irritant. If from thermal changes induced from metallic filling, it should be either removed and replaced with a non-conducting one, as gutta percha or Hill's stopping, or when a large gold filling is present on an approximate surface, I am in the habit of packing the space between the teeth with warm gutta percha, covering the metal plug entirely, allowing such covering, *if the pain ceases at once*, to remain two or three weeks, renewing it if necessary. If the metal filling is removed, it is well to dress the cavity with either creosote alone, or equal parts of creosote and tr. aconite root, placing over the applied dressing the ordinary red gutta perch temporarily. The dressing should be renewed every forty-eight hours.

Some of the worst cases of congestion of the pulp have occurred recently in my practice from the use of the oxychloride of zinc, known as German cement, and in most of them death of the pulp occurred, though every effort was made to preserve their vitality. Mechanical and chemical abrasion of teeth on grinding surfaces, with accompanying congestion of pulp, has been treated by me, successfully as follows: Place a small piece of wax carefully over the part that responds acutely to thermal changes, cover every part of the tooth with plaster-of-paris, and when it is hard, remove the wax, and into the "coffer-dam" place a few d1ops of deliquesced chloride of zinc, or a strong solution of nitric acid, allowing it to remain five or ten minutes. In many cases one application is all that is necessary. When an acid is used, an alkali should be applied in the coffer-dam before the plaster-of-paris is removed. In not a few of these cases, relief is only obtained by the use of an anæsthetic (ether,) which allows the operator, during the period of narcosis, to drill through the dentine and remove the living pulp by the nerve broach. If congestion of pulp arises from the continued use of the remedial agents previously referred, it would be well to intermit them, or if from the diseases mentioned, the indications would be for the judicious use of the alteratives and tonics.

Mal-occlusion and badly fitting clasps should at once be overcome, and we should not fail to recognize that nature here, as in every part of the body, requires rest in order to get back to normal condition, and that any interference with or disturbance of functions or nutrition is more or less expressed by structural derangement.

I have said nothing of the congestion which not unfrequently, and I might, with sadness, say, we of Philadelphia have, as a result of attempt at saving exposed pulps by the most approved methods of capping. Why we are generally unsuccessful, no one can explain as yet, though it is probably due to the *food* we eat, the *air* we breathe, the *water* we drink, or *perhaps* our inefficiency in operating.(?) In such cases, the treatment is removal of the primary irritant and application of appropriate dressings already indicated, making every effort to save the pulp by a tolerant, non-conducting filling.—*Dental Office and Laboratory.*

